# Complete aqueous defluorination of PFAS in aqueous film-forming foam (AFFF) by pulsed electrolysis with tailored potential modulation†

**DOI:** 10.1039/d4ra08214a

**Published:** 2025-03-17

**Authors:** Ziyi Meng, Teona Taseska, Madeleine K. Wilsey, Astrid M. Müller

**Affiliations:** a Materials Science Program, University of Rochester Rochester New York 14627 USA; b Department of Chemical Engineering, University of Rochester Rochester New York 14627 USA astrid.mueller@rochester.edu; c Department of Chemistry, University of Rochester Rochester New York 14627 USA

## Abstract

Per- and polyfluoroalkyl substances (PFAS) are harmful and persistent global water contaminants. AFFF, a foam-forming aqueous mixture used for firefighting, is a major source of PFAS pollution and challenging to defluorinate. We report the complete photo-assisted electrocatalytic defluorination of PFAS in AFFF with nonprecious materials. The high salt content in the aqueous LiOH electrolyte prevented foaming. Pulsed electrolysis with tailored potential modulation balanced anodic fluoride removal and the adsorption of unreacted anionic PFAS. Our findings demonstrate the effectiveness of pulsed electrocatalysis in defluorinating persistent PFAS in AFFF.

## Abstract 2

We demonstrate complete photo-assisted electrocatalytic defluorination of PFAS in AFFF using nonprecious materials. High-salt LiOH electrolyte prevents foaming, and pulsed electrolysis with tailored potential modulation optimizes PFAS defluorination.

## Graphical abstract

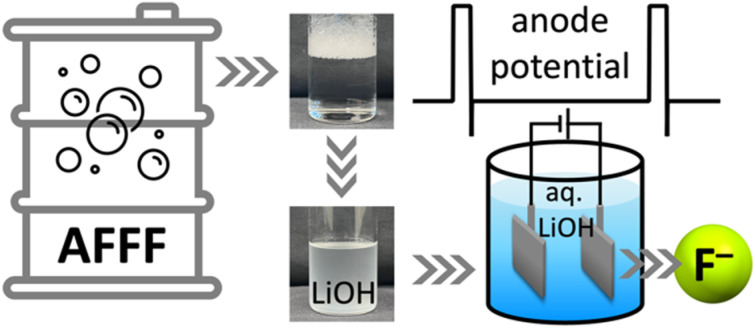

PFAS are synthetic chemicals that have been extensively used for decades in many industrial applications and consumer products.^1^ PFAS pose serious environmental and health risks due to their chronic toxicity and persistence.^1–5^ AFFF firefighting foam has widely been used for extinguishing hydrocarbon-based fuel fires at military bases, airports, petroleum refineries, chemical plants and other industrial sites.^6,7^ AFFF contains several PFAS chemicals that contribute to its stability.^6^ Because of the high toxicity of PFAS, AFFF is being phased out.^8,9^ Nevertheless, extensive use of AFFF together with PFAS persistence has left a legacy of contamination on an enormous scale.^10,11^ Therefore, efficient and affordable methods for the defluorination of PFAS in AFFF must be developed. Viable technologies must defluorinate PFAS completely, *i.e.* cleave all C–F bonds to produce fluoride.^1^ Further requirements are use of nonprecious materials only, inherent scalability, which is challenging for high-pressure based methods, low capital and operational expense, high energy efficiency, which excludes heat treatments, deployability in distributed fashion to destroy PFAS at the point of contamination or water use, and no secondary contaminated waste generation, which rules out standalone sorption methods.^1^ Ergo, electrochemical aqueous PFAS destruction has emerged as a promising strategy.^12,13^

Destruction of PFAS in AFFF by an advanced reduction process has been reported.^14^ We focus on oxidative PFAS defluorination because advanced reduction processes require oxygen-free conditions to achieve high efficiency, which is not practical on a large scale.^15^ Electrochemical oxidation processes at boron-doped diamond (BDD) electrodes have been reported for the degradation of PFAS in AFFF.^16–19^ However, BDD electrodes are cost intensive and therefore inherently not scalable.^1^

Here, we report the complete defluorination of PFAS in AFFF at [NiFe]–(OH)_2_–hydrophilic carbon fiber paper anodes by ultraviolet (UV) light assisted electrocatalysis, using pulsed electrolysis with tailored potential modulation, including a short pulse at reverse potential. This study leverages the mechanistic insights gained from the complete deep UV light assisted electrocatalytic defluorination of the PFAS perfluorooctanesulfonate (PFOS) in aqueous 8.0 M LiOH electrolyte, using only nonprecious materials and no consumed additives.^20^ We note that lithium is not depleted in our process. In previous work, we demonstrated that aqueous LiOH electrolyte outperforms other alkali metal hydroxide electrolytes by at least a factor of two in defluorination efficiency.^20^ We elucidated the role of LiOH in UV light assisted electrocatalytic PFAS defluorination and established that high concentrations of Li^+^ and OH^−^ ions are crucial for this process.^15^ Experimental evidence indicates the presence of Li–F ion pairing and competitive adsorption by hydroxide in aqueous LiOH electrolyte, both of which play a critical role in fluoride removal following C–F bond cleavage. Effective fluoride removal from the anode surface is key for preventing anode fouling, thereby enabling sustained C–F bond cleavage.^15^

We further established that while an anodic potential is necessary for C–F bond cleavage by O˙^−^ radicals—generated through deep UV photolysis of electrocatalytically produced deprotonated hydrogen peroxide—pulsed electrolysis, which involves the periodic application of potentials alternating with intervals at open-circuit potential (OCP), enhances defluorination.^15,20–22^ This enhancement results from a synergistic effect between pulsed electrolysis and the use of aqueous LiOH electrolyte, which facilitates fluoride removal at the anode. Due to the electrostatic attraction between opposite charges, negatively charged fluoride ions and Li–F ion pairs with their high dipole moment can only detach from the electrode surface and diffuse into the bulk electrolyte when the applied potential switches from anodic to OCP.^15^ Incorporating a brief cathodic potential pulse after the longer anodic pulse, which is essential for C–F bond cleavage, further enhances fluoride removal through electrostatic repulsion at the briefly negatively charged electrode. This promotes the diffusion of Li–F ion pairs away from the electrode and into the bulk electrolyte during the OCP intervals. Understanding how pulse train parameters—such as polarity, amplitude, and pulse duration—affect defluorination, based on established mechanistic insights, is crucial for optimizing AFFF defluorination efficiency.

We chose [NiFe]–(OH)_2_ because this catalyst is nonprecious, and it is a stable and efficient alkaline water oxidation catalyst,^23^ which regeneratively produces the oxidants for C–F bond cleavage.^20^ Laser synthesis enables the preparation of surfactant-free nanomaterials with precisely controlled surface chemistries,^24^ which are needed to answer fundamental scientific questions. We used pulsed laser in liquid synthesized [NiFe]–(OH)_2_ nanosheets deposited on hydrophilic carbon fiber paper electrode supports as anodes (experimental details are in the ESI†). Nanosheets exhibited layered double hydroxide structure, evident from X-ray diffraction (XRD) and scanning electron microscopy (SEM) analysis (Fig. 1). The XRD peaks were broadened, indicating small crystallite size and the presence of stacking faults, including turbostratic disorder in the hydrotalcite-like structure^25,26^ (Fig. 1a and b), consistent with prior results for laser-synthesized [NiFe]–(OH)_2_ nanosheets.^27^

**Fig. 1:**
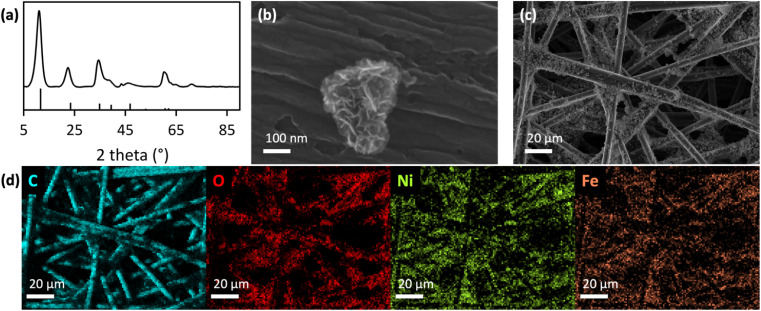
(a) XRD data of laser-made [Ni_0.75_Fe_0.25_]–(OH)_2_ nanosheets (top), with hydrotalcite powder XRD pattern^25^ (bottom). (b and c) SEM images, and (d) SEM-EDX maps of integrated anodes on hydrophilic carbon fiber paper.

Hydrophilic carbon fiber paper served as nanocatalyst support. We employed a rapid eco-friendly oxygenation process to render carbon fiber paper hydrophilic,^28^ which is needed for use in aqueous media. The carbon fiber paper had a high surface area of 468 cm^2^ per geometric cm^2^, as derived in previous work,^20^ to enhance electrocatalysis compared to a flat electrode support.^28^ We observed a uniform distribution of [NiFe]–(OH)_2_ nanocatalysts within the three-dimensional structure of the carbon fibers, effectively utilizing the high internal surface area of the electrode support (Fig. 1c and d). Analysis of the elemental composition of the laser-made [NiFe]–(OH)_2_ nanosheets by energy-dispersive X-ray spectroscopy (EDX) showed a Ni : Fe ratio of 3 : 1.

A major obstacle for the destruction of PFAS in AFFF is its exceptional foam-forming ability.^29^ AFFF is designed to create foam ‘blankets’ on the surface of burning fuel to contain flammable vapors, extinguish the fire and prevent reignition.^6^ However, in the context of PFAS defluorination, this foaming poses significant challenges for electrocatalytic C–F bond cleavage because electrochemistry requires liquid wetting of the electrode. A foam is not a liquid, but a two-phase system of gas (air) bubbles dispersed in thin liquid films. Therefore, foaming must be suppressed for efficient electrochemical destruction of PFAS in AFFF. Our electrocatalysis in aqueous 8.0 M LiOH electrolyte effectively prevented foaming of Chemguard 3% AFFF C306-MS-C (Fig. 2a), presumably due to foam bubble collapse and defoaming by the high salt concentration of the electrolyte.^30^

**Fig. 2:**
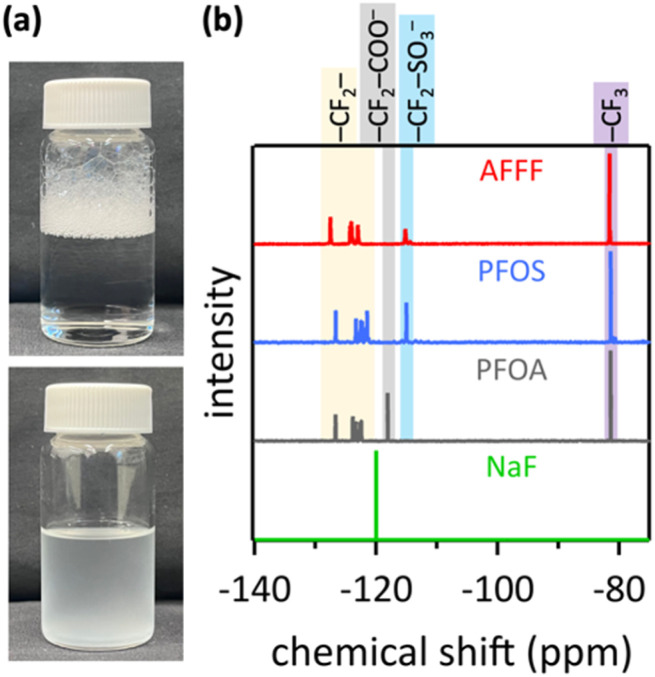
(a) Photographs of AFFF in water (top) or aqueous LiOH electrolyte (bottom) after the same agitation. (b) NMR spectra of aqueous AFFF, PFOS, PFOA, and fluoride standard, with peak assignments according to ref. 38–40.

Many different AFFF formulations exist, containing mixtures of surfactants, multiple PFAS, and proprietary substances,^31–34^ in which the PFAS identity and concentration are typically not publicly known.^35^ We determined the total fluorine content of this study's AFFF by ^19^F-NMR (Fig. 2b) because NMR detection is adequate at the concentrations used here, and liquid chromatography-tandem mass spectrometry (LC-MS/MS) methods, such as US EPA Method 1633,^36^ are very time-consuming, with wait times for results from approved laboratories of several weeks.^37^ Quantification using a sodium fluoride standard and PFOS and PFOA solutions with known concentrations showed that aqueous solutions of 1 mL AFFF added to 40 mL liquid, as used for most electrocatalysis experiments, contained 494 ppm total fluorine (experimental details are in the ESI†).

The peaks at −81 ppm in the spectra of PFOS and PFOA (Fig. 2b) were attributed to the terminal alkyl–CF_3_ fluorine nuclei, which were more exposed to the magnetic field and thus more de-shielded compared to other fluorine nuclei in the perfluorinated alkyl chain.^38,39^ The peaks in the chemical shift range of −121 to −126 ppm are attributable to CF_2_ fluorines along the perfluorinated alkyl chain, located between the terminal CF_3_ and the carbon next to the functional head group. These CF_2_ fluorines resonate at increasingly higher frequencies as they are located closer to the functional group.^38,39^ For PFOS, the peak at −114 ppm was assigned to the alkyl–CF_2_ fluorines that are next to the sulfonate group.^38^ For PFOA, the peak at −119 ppm was attributed to the alkyl–CF_2_ fluorines next to the carboxylic acid group.^40^

We quantified fluoride after AFFF defluorination by fluoride ion selective electrode (ISE) measurements. Fluoride ISEs are widely used for fluoride detection, especially in aqueous solutions, because of their high sensitivity and selectivity, with broad applications in fluoride quantification in water, industrial processes, analytical chemistry laboratories, and dental products.^41–44^ The generally accepted detection limit of the fluoride ISE is at 0.1 ppm, while the upper limit of detection is a saturated fluoride solution.^45^ The reported precision of the fluoride ISE is 0.8% for fluoride concentrations ranging from 10^−1^ to 10^−4^ M, equivalent to 1900 to 1.9 ppm.^46^ To validate the linearity, reproducibility, and experimental error of fluoride ISE detection in this study, we conducted triplicate standard measurements at six relevant concentrations (1–25 mM NaF) in both water and aqueous 8.0 M LiOH solution. We utilized potassium acetate buffer to neutralize the hydroxide ions in the strongly alkaline LiOH electrolyte to eliminate interference from hydroxide ions in the fluoride ISE measurements, in keeping with standard practice.^47,48^ We observed a linear correlation between ISE-detected fluoride and known fluoride concentrations in both water and aqueous 8.0 M LiOH solution (Fig. S1†), confirming the suitability of fluoride ISE measurements for the fluoride quantification of this study. We conducted analysis of variance (ANOVA) with post-hoc analysis to evaluate the significance of differences and obtained *p*-values below 5 × 10^−23^. This indicates that the measured differences in fluoride concentrations, determined by fluoride ISE quantification, are statistically significant, as the values are well below the widely accepted threshold of 0.05, beyond which differences are considered insignificant.^49^ Similarly, post-hoc analysis using Tukey's test^50^ confirmed that the data showed statistically significant differences.

We tailored the potential modulation in pulsed electrolysis with respect to the pulse duration and the magnitude of the applied and reverse potential, as well as the time at OCP, to understand and optimize the defluorination of PFAS in AFFF. In pulsed electrolysis, a constant applied potential is maintained for a brief time interval of seconds to minutes (ON time), followed by a switch to OCP for time durations on the order of minutes,^51^ to allow the boundary layer to re-establish equilibrium.^52^ We found in prior work that pulsed electrolysis facilitates fluoride desorption through Li–F ion pairing, competitive hydroxide ion adsorption, and the diffusion of Li–F ion pairs from the anode surface to the bulk electrolyte.^15^ Here, we added a short reverse polarity pulse after anodic C–F bond cleavage to aid in fluoride removal from the anode by electrostatic repulsion. The magnitude and the duration of this reverse potential pulse affect both fluoride repulsion and the adsorption of unreacted anionic PFAS.

We observed complete defluorination of PFAS in AFFF-containing aqueous 8.0 M LiOH electrolyte (with 247 ppm total fluorine, Fig. S2†), electrocatalyzed by laser synthesized [Ni_0.75_Fe_0.25_]–(OH)_2_ nanosheets on hydrophilic carbon fiber paper anodes, with deep UV (254 nm) light irradiation and stagnant electrolyte (Fig. 3). In our previous work on PFOS, we established that a thick electrochemical double layer, achieved by not stirring the aqueous LiOH electrolyte, is required for efficient defluorination.^20^ In general, increasing the electrolyte agitation (*e.g.* by stirring) increases the convectional mass transport in the liquid, which decreases the thickness of the electrochemical double layer.^53^ Optimum potential modulation was 30 s at *E*_ON_ = 1.6 V_RHE_, followed by 1 s at *E*_rev_ = −1.74 V_RHE_, followed by 6 min at OCP, for 120 cycles. Increasing the number of cycles to 150 also resulted in complete defluorination, albeit consuming more energy. Conversely, decreasing the number of cycles to 90 or 60 reduced defluorination linearly, reaching only 49% at 60 cycles (Fig. S3†). The highest defluorination was obtained with lower concentrations of AFFF in aqueous 8.0 M LiOH (Fig. 3a), consistent with a microenvironment process, as found for PFOS.^20^ Pulsed electrolysis completely defluorinated AFFF PFAS with concentrations of ≤13 mM total fluorine in solution. At higher AFFF concentrations, the defluorination yield decreased linearly, in line with results for PFOS.^20^

**Fig. 3:**
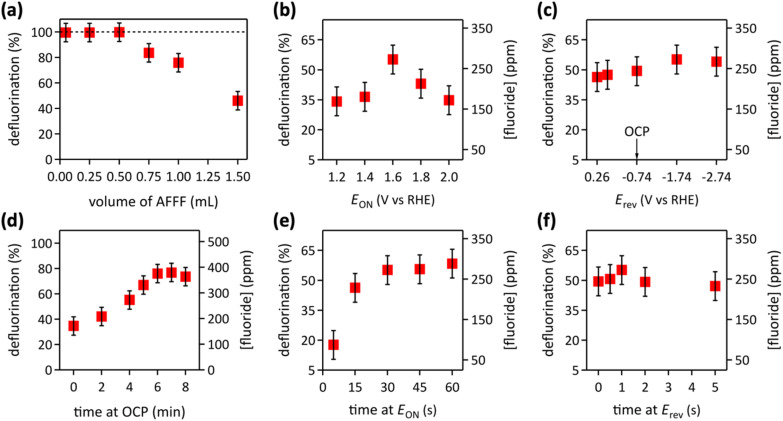
(a) Defluorination as a function of AFFF volume added to 40 mL electrolyte, using 120 pulsed electrocatalysis cycles (1 cycle = 30 s at *E*_ON_ = 1.6 V_RHE_, followed by 1 s at *E*_rev_ = −1.74 V_RHE_, followed by 6 min at OCP). The dashed line indicates 100% defluorination. (b–f) Defluorination of 1 mL AFFF in 40 mL electrolyte, with 120 cycles of pulsed electrolysis, with varied potential modulation. (b) Variation of *E*_ON_; 1 cycle = 30 s at varied *E*_ON_, followed by 1 s at *E*_rev_ = −1.74 V_RHE_, followed by 4 min at OCP. (c) Variation of *E*_rev_; 1 cycle = 30 s at *E*_ON_ = 1.6 V_RHE_, followed by 1 s at varied *E*_rev_, followed by 4 min at OCP. (d) Variation of time at OCP; 1 cycle = 30 s at *E*_ON_ = 1.6 V_RHE_, followed by 1 s at *E*_rev_ = −1.74 V_RHE_, followed by varied time at OCP. (e) Variation of time at *E*_ON_; 1 cycle = varied time at *E*_ON_ = 1.6 V_RHE_, followed by 1 s at *E*_rev_ = −1.74 V_RHE_, followed by 4 min at OCP. (f) Variation of time at *E*_rev_; 1 cycle = 30 s at *E*_ON_ = 1.6 V_RHE_, followed by varied time at *E*_rev_ = −1.74 V_RHE_, followed by 4 min at OCP. All data were collected with deep UV light irradiation of the stagnant aqueous 8.0 M LiOH electrolyte.

The optimum applied potential for AFFF PFAS defluorination in aqueous 8.0 M LiOH electrolyte was 1.6 V_RHE_ (Fig. 3b), resulting from a tradeoff of product formation in alkaline water oxidation, based on the thermodynamic potentials of the water–oxygen redox system at the electrolyte pH of 14.9, where the generation of the desired deprotonated hydrogen peroxide, HOO^−^, is maximized.^20^ The oxidant O˙^−^ arises from HOO^−^ by 254 nm light irradiation.^54^ Electrocatalytic alkaline water oxidation regenerates the C–F bond cleaving oxidant O˙^−^ within the anode microenvironment.^20^ The optimum reverse potential was −1.74 V_RHE_ (Fig. 3c). More negative potentials did not improve defluorination and lowered the energy efficiency. Highest defluorination was obtained at a time at OCP of 6 min (Fig. 3d), in line with the previously observed optimal time at OCP of 5 min for PFOS defluorination.^20^ An ON time at 1.6 V_RHE_ of at least 30 s was needed for highest defluorination (Fig. 3e). Longer ON times led to similar defluorination, but consumed more energy, whereas shorter ON times caused less efficient defluorination. The optimal duration of the reverse potential pulse was 1 s (Fig. 3f). The duration of the reversed polarity pulse forms a tradeoff between fluoride repulsion and PFAS adsorption at the anode. PFAS are anionic in the alkaline electrolyte.^1^ PFAS anions can adsorb at oxygenated carbon surfaces,^55,56^ such as the hydrophilic carbon fiber paper of this work.^20^ At reversed polarity pulse durations of less than 1 s, electrostatic fluoride repulsion appeared insufficient. Generated fluoride can block PFAS adsorption sites, fouling the anode. Conversely, at longer negative potential pulse durations, the negative charges at the electrode impeded PFAS adsorption by repelling the PFAS anions. At optimal conditions (Fig. 3a), the operational electrical energy required to completely defluorinate 1 m^3^ of typical AFFF runoff water^10^ is estimated as 68.2 kW h (details are in the ESI†). This is more efficient than existing methods, including incineration, γ-irradiation, sonolysis, non-thermal plasma treatment, and electrooxidation at BDD electrodes, as demonstrated by a recently published detailed technoeconomic analysis.^1^

Anodes composed of laser synthesized [Ni_0.75_Fe_0.25_]–(OH)_2_ nanosheets on hydrophilic carbon fiber paper provide well-defined surface characteristics essential for addressing key scientific questions. However, these anodes require labor-intensive fabrication and specialized laser equipment, limiting their widespread practicality. While the nonprecious, laser-synthesized [Ni_0.75_Fe_0.25_]–(OH)_2_ nanosheets on hydrophilic carbon fiber paper anodes remain more cost-effective than BDD anodes, even considering the laser synthesis process,^20^ lower-cost electrodes are necessary to ensure the scalability and feasibility of this technology. To address this, we utilized the widely available commercial Nifethal 70 alloy (nominal Ni/Fe: 70/30) as the anode material. However, industrial bulk materials often contain impurities,^57^ complicating scientific analysis. Nevertheless, we applied Nifethal 70 alloy under the electrocatalysis conditions optimized for the well-defined laser-synthesized [Ni_0.75_Fe_0.25_]–(OH)_2_ nanosheets on hydrophilic carbon fiber paper anodes to demonstrate the practicality of this process for AFFF defluorination.

Under anodic polarization in a strong aqueous base, first-row transition metals undergo conversion at the solid–liquid interface into their respective metal (oxy)hydroxides,^23^ indicating the potential for *in situ* formation of NiFe(O)OH species on Ni_0.70_Fe_0.30_ alloy surfaces. These NiFe(O)OH species resemble the catalyst resting state of laser-synthesized [Ni_0.75_Fe_0.25_]–(OH)_2_ material during water oxidation, which has been identified as NiFe(O)OH.^58^ To facilitate the *in situ* formation of an active catalyst, Nifethal 70 alloy anodes were conditioned at 2.0 V_RHE_ in aqueous 8.0 M LiOH for 1 h. This process *in situ* generated a surface-active NiFe(O)OH material, enabling the Nifethal 70 alloy to function as a water oxidation catalyst in a similar manner to the laser-synthesized [Ni_0.75_Fe_0.25_]–(OH)_2_ nanosheets.

SEM-EDX analysis of the Nifethal 70 alloy wire revealed the expected elements—Ni and Fe—along with Al and Si impurities (Fig. S4†), which are common in industrial bulk alloy materials.^57^ SEM data collected before and after conditioning showed the formation of a brighter nanomaterial on the darker Nifethal 70 wire surface (Fig. S4 and S5†). In SEM imaging, a brighter appearance indicates less conductivity, consistent with the formation of semiconducting NiFe(O)OH^59,60^ on the metallic alloy surface. Additionally, the increased oxygen content observed in EDX data of conditioned Nifethal 70 compared to the unconditioned material (Fig. S4 and S5†) supports the formation of surface NiFe(O)OH species, consistent with findings in the alkaline water oxidation literature.^23^ Furthermore, EDX data showed an increase in carbon content from pre-conditioning to post-conditioning and post-electrocatalysis, likely due to interlayer carbonate incorporation into NiFe(O)OH, consistent with reported findings.^27,61^ The carbonate originates from ambient carbon dioxide dissolution speciation in the strong aqueous base.^62^ After 13 h of UV-light-assisted AFFF defluorination electrocatalysis in aqueous 8.0 M LiOH, the *in situ* formed catalyst material remained visible on the Nifethal 70 alloy surface (Fig. S6†). The conditioned Nifethal 70 alloy anode enabled AFFF defluorination in aqueous 8.0 M LiOH with high stability, maintaining 98% defluorination efficiency for over 80 h (Fig. 4). The process required virtually the same estimated operational energy as the scientifically well-defined anode, as deep UV light irradiation accounts for the majority of energy consumption (see ESI† for details).

**Fig. 4:**
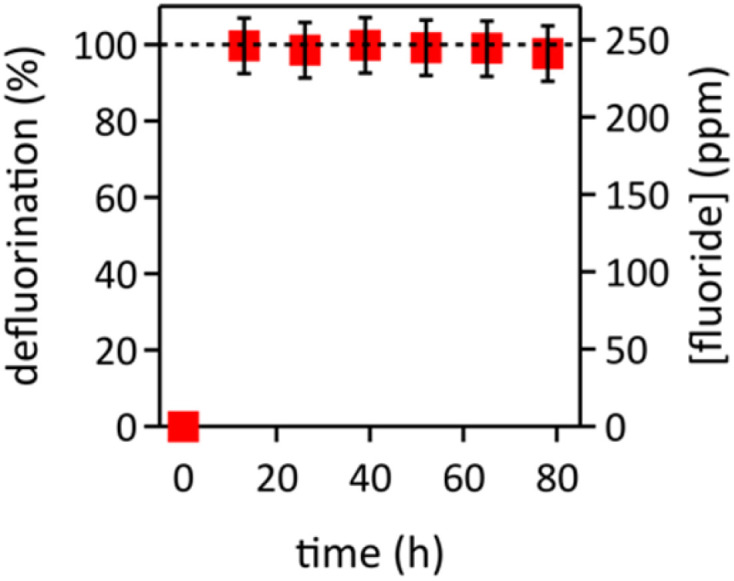
Defluorination of 0.5 mL AFFF in stagnant aqueous 8.0 M LiOH electrolyte, with deep UV light irradiation and 120 cycles of pulsed electrolysis (1 cycle = 30 s at *E*_ON_ = 1.6 V_RHE_, followed by 1 s at *E*_rev_ = −1.74 V_RHE_, followed by 6 min at OCP). Electrolyte replacement occurred every 13 h.

In summary, we achieved the complete electrocatalytic defluorination of PFAS in AFFF using UV light assisted pulsed electrolysis, without consuming bisulfate or other auxiliary chemical agents. The high salt concentration in the aqueous LiOH electrolyte prevented AFFF foaming, facilitating PFAS defluorination. We employed nonprecious laser synthesized [Ni_0.75_Fe_0.25_]–(OH)_2_ nanosheets on hydrophilic carbon fiber paper as anodes. Pulsed electrolysis with tailored potential modulation included a short pulse at reverse potential that balanced the repulsive fluoride removal from the anode surface with PFAS adsorption. The use of industrial Nifethal 70 alloy as an anode material, with *in situ* formed NiFe(O)OH species on the surface, demonstrates the practicality of the process. Our findings highlight the effectiveness of pulsed electrocatalysis and aqueous LiOH electrolyte in breaking down persistent PFAS compounds in AFFF, towards scalable energy efficient water treatment for PFAS abatement.

## Data availability

The data supporting this article have been included as part of the ESI.†

## Author contributions

Z. M.: investigation, formal analysis, visualization, and writing – original draft; T. T.: validation, formal analysis, visualization, and writing – original draft; M. K. W.: investigation, validation, and writing – original draft; A. M. M.: supervision, conceptualization, funding acquisition, and writing – review & editing.

## Conflicts of interest

There are no conflicts to declare.

## Supplementary Material

RA-015-D4RA08214A-s001
