# Supplementary material for: Complete aqueous defluorination of PFAS in aqueous film-forming foam (AFFF) by pulsed electrolysis with tailored potential modulation
Source: RSC Adv. 2025 Mar 17;15(11):8287–92. doi: 10.1039/d4ra08214a (PMC11912562; doi:10.1039/d4ra08214a)
Supplement: RA-015-D4RA08214A-s001 [file RA-015-D4RA08214A-s001.pdf]

Supplementary Information (SI) for RSC Advances.
This journal is © The Royal Society of Chemistry 2025

Supplementary Information

Complete Aqueous Defluorination of PFAS in Aqueous Film-Forming Foam (AFFF) by Pulsed Electrolysis with Tailored Potential Modulation

Ziyi Meng,[a] Teona Taseska,[b] Madeleine K. Wilsey,[a] and Astrid M. Müller*[abc]

[a] *Materials Science Program, University of Rochester, Rochester, New York, 14627, USA.*
[b] *Department of Chemical Engineering, University of Rochester, Rochester, New York, 14627, USA.*
[c] *Department of Chemistry, University of Rochester, Rochester, New York, 14627, USA.*

* *E-mail: astrid.mueller@rochester.edu*

Experimental Section

All chemicals were used as received. Deionized water was obtained from a Thermo Scientific Barnstead Smart2Pure Pro UV/UF 15 LPH Water Purification System and had a resistivity of ≥17.5 MΩ • cm. All experiments were carried out at room temperature and under ambient air. Error bars represent the standard deviations from triplicate measurements. Data analysis and graphing were performed using Igor Pro 8.04 (Wavemetrics) unless otherwise specified.

Catalyst Preparation

The process of making carbon fiber paper (FuelCellStore, AvCarb MGL190) hydrophilic is described elsewhere.[1] Briefly, as-purchased carbon fiber paper (FuelCellStore, AvCarb MGL190, 78 % porosity) was functionalized by sonication in a 1.0 M aqueous sodium dodecyl sulfate solution, followed by electrooxidation in a 0.1 M pH 8.7 aqueous $KHCO_3$ electrolyte at 1.63 V vs. Ag/AgCl for 20 min. Electrodes had geometric dimensions of 3.0 cm (length) × 3.0 cm (width), resulting in a total geometric electrode area of 9.0 cm^2.

The details for the synthesis of the laser-made nickel–iron-layered double hydroxide nanocatalyst are described elsewhere.[2] In short, we used a 10 Hz Q-switched Nd:YAG laser (Spectra-Physics Quanta-Ray LAB-190) with 8 ns pulses of 90 mJ energy and a wavelength of 355 nm to irradiate a suspension of iron powder (Alfa, −200 mesh, 99+%) in a 10 mL solution of 3.0 M nickel nitrate (Alfa, 98%) in water for 60 min. After the laser synthesis, unreacted iron powder was separated from the aqueous suspension of produced $[Ni_{0.75}Fe_{0.25}]\text{-}(OH)_2$ nanoparticles using a strong magnet. The solid nanoparticulate powder was isolated through centrifugation, washed five times with water, followed by two washes with acetone (VWR). The nanopowder was dried under house vacuum. Suspensions of the nanoparticulate powder at a concentration of 2 mg mL^{-1} in water were vortexed, drop-cast onto hydrophilic carbon fiber supporting electrodes to achieve a catalyst mass loading of 444 μg $cm^{-2}{}_{geo}$, and dried under a heat lamp at 60°C in ambient air.

Nifethal 70 wire (Coil Society) with a 0.32 mm diameter was used to fabricate alloy anodes. The wire was coiled ten times around a 3 cm × 1 cm aluminum template in a single direction, forming a rectangular-shaped structure with parallel wires. After carefully removing the aluminum template, the wire framework was gently flattened. Additional Nifethal wire was then woven perpendicularly through the framework in an alternating over-and-under pattern, creating a tight, grid-like configuration with a geometric electrode area of 3.0 cm^2. A 5 cm straight wire segment was left protruding from the structure to serve as the connection point for the potentiostat lead.

Nifethal 70 anodes were conditioned in aqueous 8.0 M LiOH at 2.0 V_{RHE} for 1 h. Following this conditioning, the wires were rinsed with deionized water, washed with acetone, and then thoroughly rinsed again with deionized water. The wires were then dried using dry nitrogen gas from liquid nitrogen boil-off.

Preparation of Solutions

The aqueous 8.0 M LiOH electrolyte was prepared by adding 19.1 g of lithium hydroxide monohydrate (Thermo Scientific, 98%) in a 100 mL volumetric flask. The flask was then filled with deionized water to the 100 mL mark and mixed until fully dissolved. Solutions were prepared by placing 0.05, 0.25, 0.5, 0.75, 1.00, or 1.50 mL of AFFF (Chemguard 3% AFFF C306-MS-C),

respectively, in a 50 mL Falcon tube, to which 40 mL of electrolyte was added. The AFFF-containing solutions were gently inverted up and down to mix the solution evenly, then left undisturbed for 30 min to ensure proper mixing and dissolution. A Sartorius A 120 S analytical or a Mettler Toledo AT201 analytical balance were used to weigh chemicals.

Physical Characterization

XRD data were acquired using a Rigaku XtaLAB Synergy-S diffraction system equipped with a HyPix-6000HE HPC detector at room temperature. A PhotonJet-S microfocus source was operated at 50 kV and 1 mA to generate Cu Kα radiation with wavelength of 1.54184 Å. Two combination ω-φ "Gandolfi" scans were performed, each for 300 s: (1) ω from –62.00 to 31.00 degrees and φ rotated through 720 degrees, at θ = –42.127 and κ = 70.000 degrees; and (2) ω from –31.00 to 61.00 degrees and φ rotated through 720 degrees, at θ = 40.877 and κ = –70.00 degrees. The sample-to-detector distance was 34 mm. A light coating of viscous oil was used to secure the $[Ni_{0.75}Fe_{0.25}]\text{-}(OH)_2$ powder sample to a Nylon loop (0.1 mm ID).

SEM imaging of anodes was performed at UR-Nano, using a Zeiss Auriga scanning electron microscope, equipped with a Schottky field emission emitter, and operated at 20.00 kV with a working distance of 5.0 mm. EDX spectroscopy data were obtained using a SEM-integrated EDAX Octane elect plus with silicon drift detector spectrometer. Double sided carbon tape was used to adhere the anodes to sample stubs.

Electrocatalysis

Electrochemical investigations were performed in a standard three-electrode single-compartment 50 mL electrochemical cell, using a Bio-Logic, 8-slot VSP3e potentiostat. The cell was fitted with a Teflon lid that remained above the liquid surface. The cell included a flat quartz window for ultraviolet light illumination (254 nm lamp, 40 mW cm^{-2}). Anodes consisted of laser-made $[Ni_{0.75}Fe_{0.25}]\text{-}(OH)_2$ nanosheets immobilized on hydrophilic carbon fiber paper, with a geometric area of 9.0 cm^2 and catalyst mass loading of 444 μg $cm^{-2}{}_{geo}$. Counter electrodes were neat hydrophilic carbon fiber paper. Alternatively, conditioned Nifethal 70 alloy with a geometric electrode area of 3.0 cm^2 and a Ni mesh (Alfa) counter electrode were used. A Pt wire pseudo-reference electrode, calibrated against a hydrogen reference electrode (Gaskatel HydroFlex®) in the electrolyte, served as the reference electrode. Platinum wire has been shown to be a suitable and stable reference electrode in various electrochemical systems.[3] The anode was positioned parallel to the cathode at a distance of 16 mm, secured by the Teflon lid, and centered in the Pyrex cell, perpendicular to the quartz window. The data were not corrected for any uncompensated resistance losses. All potentials are reported vs the reversible hydrogen electrode (RHE).

Quantification of Fluoride

The method for determining the fluoride concentration in the electrolyte post-electrocatalysis is described elsewhere.[4] Briefly, we used a fluoride ion selective electrode (Thermo Scientific Orion, 9609BNWP) and a total ionic strength adjustment buffer (TISAB II) solution. To eliminate interference from hydroxide ions in fluoride ion selective electrode measurements, potassium acetate buffer was utilized to neutralize the hydroxide ions in strongly alkaline electrolyte. Fluoride concentrations in all aqueous electrolytes after electrocatalytic AFFF defluorination were

determined by adding 1 mL of solution to 9 mL of potassium acetate buffer in a 50 mL Falcon tube, followed by the addition of 10 mL of TISAB II solution.

A series of solutions with fluoride concentrations of 25, 20, 15, 10, 5, or 1 mM were prepared by adding 2.5, 2.0, 1.5, 1.0, 0.5, or 0.1 mL of commercially available fluoride standard (Thermo Scientific, 0.10 M sodium fluoride), respectively, to a 10 mL volumetric flask that was subsequently filled to the 10 mL mark with water or aqueous 8.0 M LiOH solution and mixed well. The fluoride concentrations in these solutions were 475, 380, 285, 190, 95, or 19 ppm, respectively. Analysis of Variance (ANOVA) and post-hoc analysis was performed in Python 3.13.1. The dataset was structured as a Pandas DataFrame, where one column represented the known concentrations and the second column contained the measured values. A one-way ANOVA was conducted using the ols function from the statsmodels.formula.api module to fit the model, followed by the anova_lm function from statsmodels.api to compute the ANOVA table. The significance of the test was determined based on the p-value. Since the ANOVA results indicated statistically significant differences ($p \ll 0.05$), a post-hoc analysis was performed using Tukey's test (pairwise_tukeyhsd from statsmodels.stats.multicomp) to identify which groups differed from each other.

AFFF Foaming

Photographs of AFFF foaming, shown in Fig. 2a of the main text, were taken of 12 mL of AFFF solutions in vials that were each shaken by hand five times. The solutions were prepared by adding 1 mL Chemguard 3% AFFF C306-MS-C to 40 mL of water or aqueous 8.0 M LiOH.

Determination of Fluorine in AFFF

The total fluorine concentration of AFFF was determined by ^{19}F-NMR, recorded on a JEOL ECZL 400 MHz spectrometer. All NMR data were obtained using a 1.5 s acquisition time, a 2 s relaxation delay time, and 32 scans. Data were processed in the MestReNova (MNova) software, using baseline correction (Bernstein polynomial fit) and 1 Hz apodization. To quantify the fluorine content, the integrated area of an aqueous fluoride standard solution (Orion 940906, Thermo Scientific) was compared to that of 1 mL AFFF added to 40 mL of water. The same NMR tube was used for all samples, to exclude signal strength differences due to path length differences. The fluoride standard solution contained 0.10 M sodium fluoride, corresponding to a fluorine concentration of 1900 ppm. Potassium perfluorooctanesulfonate (PFOS) and perfluorooctanoic acid (PFOA) were used as controls. The PFOS sample consisted of 0.1688 g of potassium perfluorooctanesulfonate (Synquest Laboratories, 95 %) dissolved in 200 mL water, resulting in a PFOS concentration of 1.57 mM, corresponding to a total fluorine concentration of 504 ppm. The PFOA sample consisted of 0.1673 g of perfluorooctanoic acid (Beantown Chemical, 95 %) dissolved in 200 mL water, resulting in a PFOA concentration of 2.02 mM, corresponding to a total fluorine concentration of 515 ppm. For the sample consisting of 1 mL AFFF added to 40 mL of water, we obtained a total fluorine concentration of 494 ppm, which corresponds to a total fluorine concentration of 20,254 ppm in neat AFFF, consistent with the manufacturer's safety data sheet, stating that Chemguard 3% AFFF C306-MS-C contains 1 – 5 wt% proprietary polyfluorinated alkyl polyamide and 0.1 – 1 wt% proprietary polyfluorinated alkyl quaternary amine chloride.

^{19}F-NMR Data Analysis

Quantification of total fluorine in Chemguard 3% AFFF C306-MS-C was performed by integrating peak areas. Concentrations of aqueous solutions with known PFOS and PFOA concentrations were used to validate the ^{19}F-NMR peak integration method. Aqueous 1.57 mM PFOS solution with a calculated total fluorine concentration of 504 ppm resulted in an NMR-derived concentration of 507 ppm. Aqueous 2.02 mM PFOA solution with a calculated total fluorine concentration of 515 ppm resulted in an NMR-derived concentration of 522 ppm. This results in a relative error of 1.4%.

Estimation of Operational Energy Consumption

At laboratory scale, complete defluorination of PFAS in AFFF with 247 ppm total fluorine required 1.88×10^{-5} or 1.05×10^{-4} kWh of electrical energy for the laser-synthesized $[Ni_{0.75}Fe_{0.25}]$-$(OH)_2$ on hydrophilic carbon fiber paper or Nifethal 70 anodes, respectively. This electrical energy consumption was derived from potentiostat data. The unit ppm corresponds to mg L^{-1}. The defluorination was assisted by 40 mW cm^{-2} 254 nm light irradiation from a 4.5 W UV lamp, which consumed 0.059 kWh of energy. Thus, the total laboratory-scale electrical energy consumption was 0.059 kWh, regardless of whether the catalyst was laser-synthesized or in-situ formed at Nifethal 70 alloy. To be able to compare the energy efficiency of our process to other existing methods, we extrapolated the laboratory scale to the energy required to treat 1 m^3 of contaminated water. The mean concentration of AFFF in contaminated water is 10,000 ng L^{-1}.[5] Defluorination of PFAS with lower concentrations was more efficient with our technology (cf. Fig. 3a of the main text and ref. 4) In aqueous systems that use concentrated electrolytes with high conductivities, the maximum accepted inter-electrode distance is generally 10 cm.[6] This means that 10 electrolyzer stacks of 1 m^2 geometric area would be needed, corresponding to a geometric anode area of 10 m^2.[7] Scaling the volume up from the laboratory scale of 40 mL to 1 m^3 requires a scaling factor of 25,000. According to the manufacturer, the density of Chemguard 3% AFFF C306-MS-C is 1.02 g mL^{-1}. Therefore, the calculated energy requirement for the scaled-up AFFF electrocatalysis would amount to 9.20×10^{-6} kWh. The laboratory scale deep UV light source was used at a power of 40 mW cm^{-2} to illuminate the electrolyzer through a circular window with a diameter of 2 cm. At the laboratory scale, the calculated energy input of the UV lamp was 1.64×10^{-3} kWh. Using the scalability factor of 25,000, the deep UV LED device would consume 40.95 kWh if it operated at 100% efficiency. Compared to the laboratory scale deep UV light source, a 1 m^3 wastewater treatment system would utilize an industrial-scale deep UV light emitting diode (LED) device with a typical efficiency of 60%.[8] Factoring in the LED device efficiency, the energy required for ultraviolet irradiation at the industrial scale is calculated to be 68.2 kWh. Thus, the total operational electrical energy consumption for complete AFFF defluorination in 1 m^3 wastewater treatment system would be 68.2 kWh.

Supplementary Figures

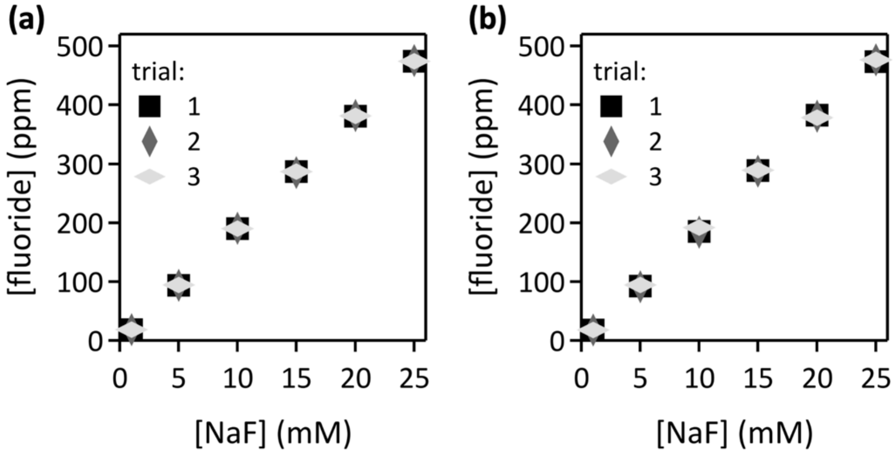

Fig. S1 Fluoride quantified by fluoride ion selective electrode measurements vs known fluoride concentrations in (a) water and (b) aqueous 8.0 M LiOH solution.

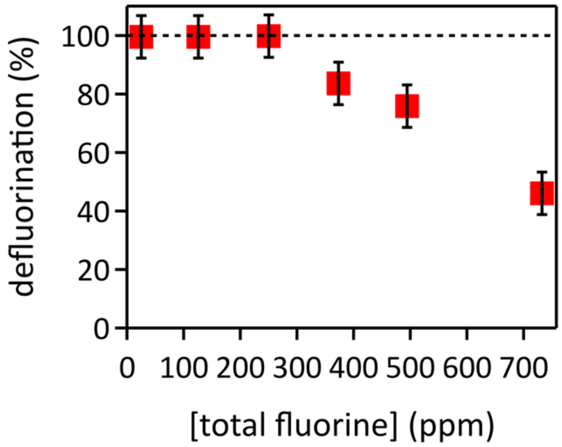

Fig. S2 Defluorination as a function of total fluorine concentration in the solution of AFFF in the electrolyte, using 120 pulsed electrocatalysis cycles (1 cycle = 30 s E_{ON} = 1.6 V_{RHE}, followed by 1 s at E_{rev} = –1.74 V_{RHE}, followed by 6 min at OCP). The dashed line indicates 100% defluorination.

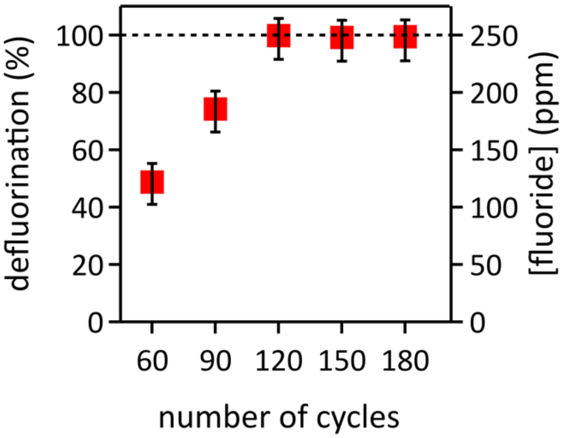

Fig. S3 Defluorination of PFAS in AFFF as a function of number of cycles (1 cycle = 30 s E_{ON} = 1.6 V_{RHE}, followed by 1 s at E_{rev} = –1.74 V_{RHE}, followed by 6 min at open circuit potential). The dashed line indicates 100% defluorination. Data were collected with deep UV light irradiation of the stagnant aqueous 8.0 M LiOH electrolyte, and 0.5 mL AFFF added to 40 mL electrolyte was used.

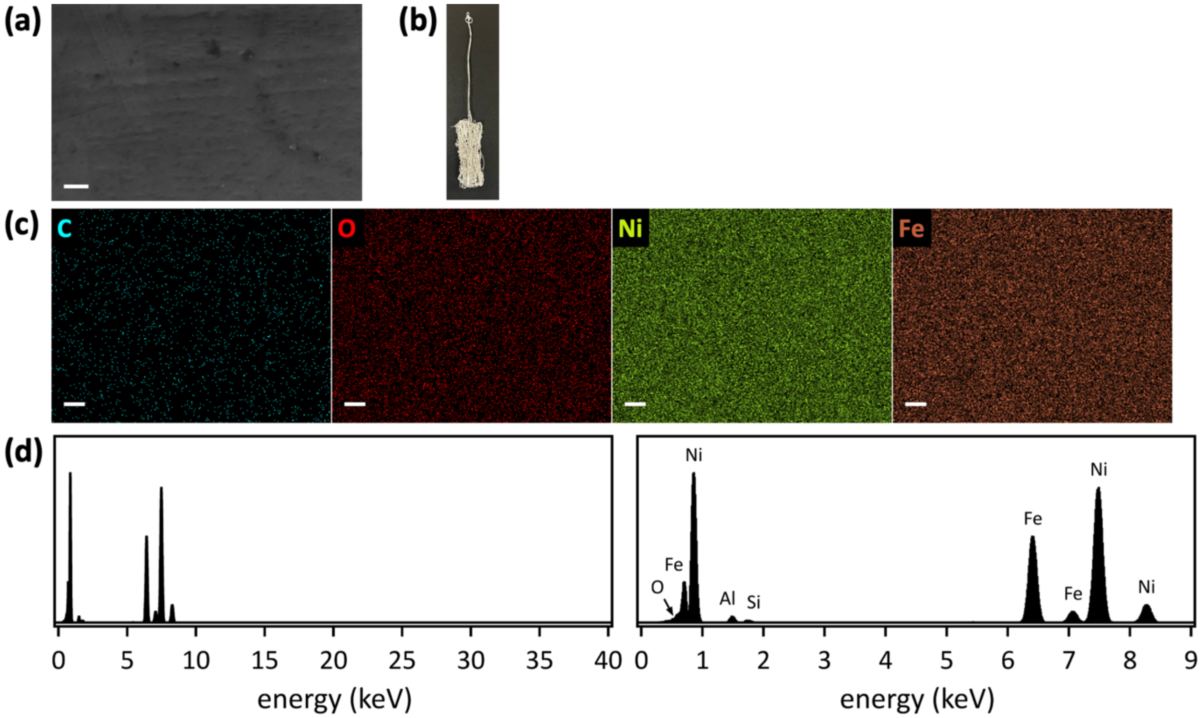

Fig. S4 (a) SEM image, (b) photograph, (c) EDX maps, and (d) EDX spectra in different energy ranges of Nifethal 70 alloy anode before conditioning. All scalebars are 400 nm.

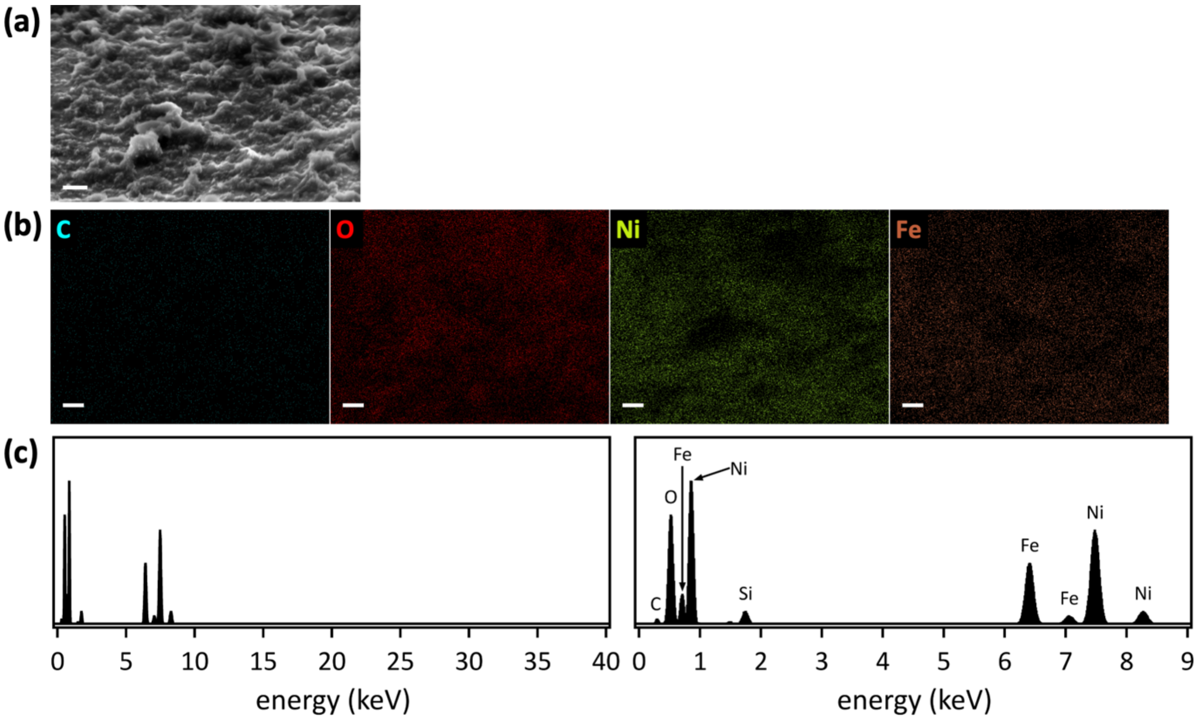

Fig. S5 (a) SEM image, (b) EDX maps, and (c) EDX spectra in different energy ranges of Nifethal 70 alloy anode after conditioning. All scalebars are 400 nm.

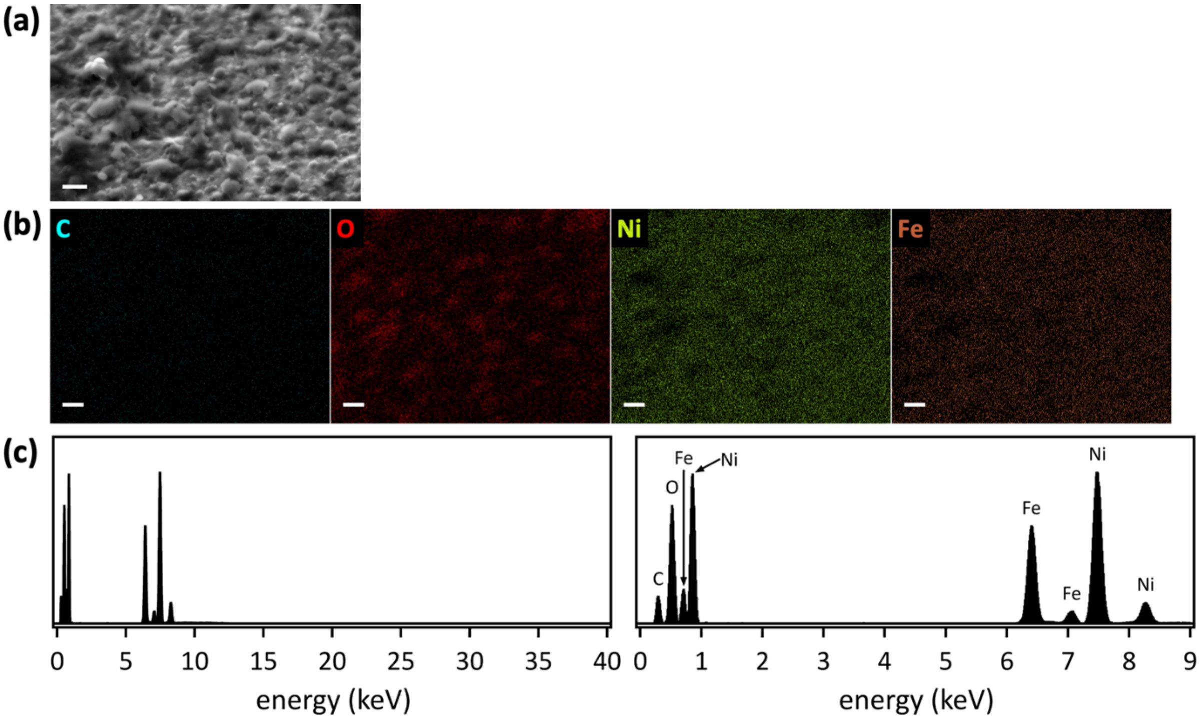

Fig. S6 (a) SEM image, (b) EDX maps, and (c) EDX spectra in different energy ranges of Nifethal 70 alloy anode after 13 h electrocatalysis. All scalebars are 400 nm.

References

1 M. K. Wilsey, K. R. Watson, O. C. Fasusi, B. P. Yegela, C. P. Cox, P. R. Raffaelle, L. Cai and A. M. Müller, *Adv. Mater. Interfaces*, 2023, **10**, 2201684.
2 B. M. Hunter, J. D. Blakemore, M. Deimund, H. B. Gray, J. R. Winkler and A. M. Müller, *J. Am. Chem. Soc.*, 2014, **136**, 13118-13121.
3 K. K. Kasem and S. Jones, *Platinum Metals Rev.*, 2008, **52**, 100.
4 Z. Meng, M. K. Wilsey, C. P. Cox and A. M. Müller, *J. Catal.*, 2024, **431**, 115403.
5 B. J. Ruyle, C. P. Thackray, C. M. Butt, D. R. LeBlanc, A. K. Tokranov, C. D. Vecitis and E. M. Sunderland, *Environ. Sci. Technol.*, 2023, **57**, 8096-8106.
6 E. Amores, J. Rodríguez and C. Carreras, *Int. J. Hydrogen Energy*, 2014, **39**, 13063-13078.
7 M. K. Wilsey, T. Taseska, Z. Meng, W. Yu and A. M. Müller, *Chem. Commun.*, 2023, **59**, 11895-11922.
8 M. Martín-Sómer, C. Pablos, C. Adán, R. van Grieken and J. Marugán, *Sci. Total Environ.*, 2023, **885**, 163963.
